# Effect of MSCs and MSC-Derived Extracellular Vesicles on Human Blood Coagulation

**DOI:** 10.3390/cells8030258

**Published:** 2019-03-19

**Authors:** Denis N. Silachev, Kirill V. Goryunov, Margarita A. Shpilyuk, Olga S. Beznoschenko, Natalya Y. Morozova, Elizaveta E. Kraevaya, Vasily A. Popkov, Irina B. Pevzner, Ljubava D. Zorova, Ekaterina A. Evtushenko, Natalia L. Starodubtseva, Alexey S. Kononikhin, Anna E. Bugrova, Evgeniy G. Evtushenko, Egor Y. Plotnikov, Dmitry B. Zorov, Gennady T. Sukhikh

**Affiliations:** 1 V.I. Kulakov National Medical Research Center of Obstetrics, Gynecology and Perinatology, Moscow 117198, Russia; d_silachev@oparina4.ru (D.N.S.); kirishgor@gmail.com (K.V.G.); tambovtsevamr@mail.ru (M.A.S.); n_morozova@oparina4.ru (N.Y.M.); e_kraevaya@oparina4.ru (E.E.K.); popkov.vas@gmail.com (V.A.P.); irinapevzner@mail.ru (I.B.P.); lju_2003@list.ru (L.D.Z.); n_starodubtseva@oparina4.ru (N.L.S.); alex.kononikhin@gmail.com (A.S.K.); annabugrova@gmail.com (A.E.B.); gtsukhikh@mail.ru (G.T.S.); 2 A.N. Belozersky Institute of Physico-Chemical Biology, Lomonosov Moscow State University, Moscow 119992, Russia; 3 Faculty of Biology, Lomonosov Moscow State University, Moscow 119234, Russia; trifonova.katerina@gmail.com; 4 Moscow Institute of Physics and Technology, Moscow 141701, Russia; 5 Emanuel Institute of Biochemical Physics, Russian Academy of Sciences, Moscow 119334, Russia; 6 Faculty of Chemistry, Lomonosov Moscow State University, Moscow 119992, Russia; evtushenko@enzyme.chem.msu.ru; 7 Department of Obstetrics, Gynecology, Perinatology and Reproductology, Institute of Professional Education, First Moscow State Medical University Named after I.M. Sechenov, Moscow 119992, Russia

**Keywords:** mesenchymal stem cells, extracellular vesicles, thrombosis, heparin, procoagulant proteins

## Abstract

Mesenchymal stem cells (MSCs) have emerged as a potent therapeutic tool for the treatment of a number of pathologies, including immune pathologies. However, unwelcome effects of MSCs on blood coagulation have been reported, motivating us to explore the thrombotic properties of human MSCs from the umbilical cord. We revealed strong procoagulant effects of MSCs on human blood and platelet-free plasma using rotational thromboelastometry and thrombodynamic tests. A similar potentiation of clotting was demonstrated for MSC-derived extracellular vesicles (EVs). To offer approaches to avoid unwanted effects, we studied the impact of a heparin supplement on MSC procoagulative properties. However, MSCs still retained procoagulant activity toward blood from children receiving a therapeutic dose of unfractionated heparin. An analysis of the mechanisms responsible for the procoagulant effect of MSCs/EVs revealed the presence of tissue factor and other proteins involved in coagulation-associated pathways. Also, we found that some MSCs and EVs were positive for annexin V, which implies the presence of phosphatidylserine on their surfaces, which can potentiate clot formation. Thus, we revealed procoagulant activity of MSCs/EVs associated with the presence of phosphatidylserine and tissue factor, which requires further analysis to avoid adverse effects of MSC therapy in patients with a risk of thrombosis.

## 1. Introduction

Mesenchymal stem cells (MSCs) have emerged as a potent therapeutic tool in reparative cell technologies with a high capacity to modulate immune responses associated with their minimal immunogenicity [1]. These specific immunomodulatory effects of MSCs could be beneficial for the treatment of sepsis and other inflammatory diseases [2]. It is believed that these and other therapeutic effects of MSCs are realized through paracrine mechanisms, accompanied by their well-determined production of extracellular vesicles (EVs) [3], which promote similar immunomodulatory potential as the MSCs themselves [4,5,6]. Both autologous and allogeneic human MSCs are currently being broadly tested in about 500 clinical trials worldwide, and severe side effects have been rarely observed [7]. One of the few identified complications is the risk of therapy-induced thrombosis, reported in several patients [8,9,10,11]. A recent clinical trial revealed that intravenous infusion of allogeneic adipose-derived MSCs exerted mixed pro- and anti-inflammatory as well as procoagulant effects during human endotoxemia [12]. Procoagulant activity was also observed for other types of transplanted cells, such as pancreatic islets and hepatocytes [13,14]. It was found that procoagulant activity could be linked to tissue factor (TF) expression [8,13,14,15,16]. Notably, long-lasting MSC culturing leads to a remarkably high expression of TF on the cells’ surface, which could increase the risk of clot formation [8,17]. In addition to TF, there is another procoagulation factor, phosphatidylserine, which, after transitioning from the inner to the outer leaflet of the cell membrane, creates a platform to build up the thrombin-activating complex [18]. It can also be assumed that EVs isolated from MSC-conditioned medium can contain TF and/or external phosphatidylserine and therefore affect blood hemostasis as well.

Today, at least two methods for attenuating the procoagulant potency of MSCs have been proposed. The first approach is direct blocking of TF moiety on the MSC surface using specific antibodies, but this protocol seems difficult to implement in clinical applications [8,19]. The second approach proposes the use of anticoagulation therapy, such as heparin, to target soluble blood coagulation factors. In this context, clinical protocols imply concomitant administration of MSCs and heparin [17,19]. This protocol of MSC transplantation with simultaneous anticoagulant treatment has demonstrated efficacy in animal models; however, it raises a number of serious issues for further translation into clinical practice, since it has significant limitations. We must admit that among the potential objects of MSC therapy, there are many patients with a complicated coagulopathic history. For example, this category of patients includes pregnant women with pathologies [20,21] and newborns with congenital sepsis [22]. Although cell therapy could be very useful for such patients [23,24], interventions that cause changes in coagulatory function could lead to dramatic circumstances for this cohort. Also, it remains unclear whether heparin binds to the MSC surface or to soluble blood coagulation factors, and what heparin dose would be effective for the attenuation of procoagulant activity of MSCs without adverse systemic effects on hemostasis. Although the use of EVs appears to be an alternative to the therapeutic use of MSCs, there is no evidence for the absence of an effect of the vesicles on coagulation. Therefore, elucidating the mechanisms and factors affecting MSC and EV coagulation properties might be very important in further applications of stem cell technologies, particularly in inflammation-associated pathologies, since it is well known that inflammation leads to the activation of blood coagulation [25].

The aim of the present study was to determine whether MSCs from umbilical cord and MSC-derived EVs display procoagulant activity and to find a possible correlation of such activity with procoagulant proteins. Moreover, we investigated how procoagulant activity could be modulated using different protocols of cell treatment.

## 2. Materials and Methods

### 2.1. Primary Culture of MSC

Fresh human umbilical cords were obtained from healthy women 25 to 30 years old (*n* = 6) who delivered healthy full-term infants by cesarean section at the V.I. Kulakov National Medical Research Center for Obstetrics, Gynecology, and Perinatology. These women had no history of infectious diseases or pregnancy complications and were confirmed to be negative for hepatitis B virus (HBV), human immunodeficiency virus (HIV), and syphilis. The research was carried out according to the World Medical Association Declaration of Helsinki and with the permission of the local ethics committee of V.I. Kulakov National Medical Research Center of Obstetrics, Gynecology, and Perinatology (Protocol No. 1 from 29 January 2015), and informed consent was obtained from all subjects.

Umbilical cords obtained after birth were washed in phosphate-buffered saline (PBS) (Paneco, Moscow, Russia) several times. After blood vessels were removed, the umbilical cords were minced into 1 cm^3^ fragments and subsequently homogenized into 1–2 mm^3^ pieces. The cells were cultivated in Dulbecco’s Modified Eagle Medium (DMEM)/F12 (Paneco, Moscow, Russia) (1:1) containing 7% fetal bovine serum (Biosera, Nuaille, France) supplemented with penicillin (100 IU/mL), streptomycin (100 μg/mL) (Gibco, NY, USA), and 2 mM L-glutamine (Paneco, Moscow, Russia) and incubated in a humidified atmosphere with 5% CO_2_ at 37 °C. The incubation medium was refreshed every 3–4 days to remove nonadherent cells. Cell growth and morphology were monitored daily under an inverted microscope. MSCs at the third passage were used in the experiments. The cells were trypsinized, centrifuged (1600× *g* for 3 min), resuspended in 10 μL of PBS, and used immediately. The cell viability was assessed by trypan blue exclusion (generally >95%). MSCs used in our study were positive for mesenchymal stem cell markers (CD73, CD90, CD105) and negative for hematopoietic cell markers (CD14, CD20, CD45, CD34) (Supplementary Figure S1).

### 2.2. Isolation of Extracellular Vesicles by Differential Centrifugation

Differential centrifugation was used for isolation of EVs from conditioned medium as described previously [26]. Supernatants were collected from conditioned medium of MSC cultures of passage 3 at 80–90% confluence (~10 × 10^6^ cells) 24 h after being refreshed with medium (DMEM/F12 containing 7% fetal bovine serum (FBS), 2 mM L-glutamine, 100 U/mL penicillin, and 100 μg/mL streptomycin). Prior to use, the culture medium was centrifuged at 108,000× *g* for 1.5 h to avoid possible contamination with EVs aroused from FBS, then supernatant was harvested, filtered using a bottle-top vacuum filter system with a pore size of 0.22 μm (Falcon, Corning, NY, USA), and used for further experiments as vesicle-free culture medium. Conditioned medium (50 mL) from confluent cultures was collected and processed using serial centrifugations to remove cells and debris (400× *g* for 10 min followed by 10,000× *g* at 4 °C for 30 min). Supernatant was used for EV isolation by ultracentrifugation at 108,000× *g* for 1.5 h at 4 °C by an Avanti JXN-30 high-speed centrifuge (Beckman Coulter Inc., Fullerton, CA, USA) with further pellet washing with phosphate buffered saline (PBS) followed by another spin at 108,000× *g* for 1.5 h to minimize protein contamination. The final EV pellet was resuspended in 10 μL of filtered PBS. Vesicle samples were stored at −80 °С. Resuspended pellet from nonconditioned culture medium passed through all centrifugations was used as an additional control sample (blank EV) to ensure that the observed effects were caused by EVs from MSCs and not by an unavoidable admixture of adventitious nanoparticles.

### 2.3. Blood Sampling

The procedure of blood collection was carried out according to the World Medical Association Declaration of Helsinki and with the permission of the local ethics committee of V.I. Kulakov National Medical Research Center of Obstetrics, Gynecology, and Perinatology (Protocol No. 1 from 4 February 2016), and informed consent was obtained from blood donors’ or newborns’ legal representatives.

Blood samples were collected from 6 healthy donors aged 28–38 years old. Donors did not receive any medication for 2 weeks prior to the investigation.

To assess whether heparin treatment in vivo reduces the procoagulant activity of MSCs in human blood, we used the blood of term newborn infants (*n* = 7) with a high risk of thrombosis. Patients received unfractionated heparin sodium salt at the dose of 5 U/h/kg intravenously. In routine blood sampling through a peripheral catheter to monitor the concentration of heparin in the blood (anti-Xa assay), part of the blood was used to analyze the procoagulant effects of MSCs in vitro. The blood samples were collected 0.5–1 h after heparin administration.

Blood was drawn into vacuum tubes (Monovette, Sarstedt, Germany) with 106 mM sodium citrate buffer (pH 5.5) at a 9:1 blood:anticoagulant volume ratio. The blood was obtained during fasting and the analysis was performed within 30 min. Whole blood was used for rotational thromboelastometry. Part of the blood sample was centrifuged at 1600× *g* for 16 min to obtain platelet-poor plasma. This plasma was repeatedly processed by centrifugation at 10,000× *g* for 5 min to obtain platelet-free plasma (PFP), which was used for thrombodynamics and anti-Xa assay.

### 2.4. Rotational Thromboelastometry (ROTEM)

Measurements were performed on a ROTEM^®^ delta analyzer (Pentapharm, Munich, Germany), which assessed the kinetics and quality of clot formation and clot lysis in real time. Within 30 min after taking a blood sample, a nonactivated rotational thromboelastometric (NATEM) test was performed. In brief, 10 μL of PBS, EVs, or suspended cells were added to 1 mL of whole blood. Final cell concentrations were 5 × 10^3^, 1 × 10^4^, and 5 × 10^4^ cells/mL, and the final EV concentration was 4.3 × 10^10^ vesicles/mL. Then, 300 µL of the sample was transferred to a prewarmed (37 °C) ROTEM mini-cup, followed by supplementation with 20 µL of 0.2 M CaCl_2_. Parameters assessed were the clotting time (CT), clot formation time (CFT), maximum clot firmness (MCF), and α-angle. CT was defined as the period of time from the start of analysis until the start of clot formation, normally until a 2 mm amplitude was reached. Clot formation time was classified as the period until a 20 mm amplitude was reached. The α-angle was defined as the angle between the center line and a tangent to the curve through the 2 mm amplitude point, which was at the end of the CT. MCF was defined as the maximal amplitude in the trace, which reflects the absolute strength of the fibrin and platelet clot.

### 2.5. Thrombodynamics Assay

Thrombodynamics (TD) assay was performed using the Thrombodynamics Analyzer (HemaCore LLC, Moscow, Russia) according to the previously described protocol [27]. This assay is based on videomicroscopic observation of fibrin clot propagation in a nonstirred layer of plasma activated by immobilized tissue factor. PFP obtained from 1 mL of whole blood was mixed with either cells (5 × 10^3^, 1 × 10^4^, or 5 × 10^4^ cells) or EVs (4.3 × 10^10^, 4.3 × 10^9^, or 4.3 × 10^8^ vesicles) or an equal volume of PBS. The sample (120 µL of the mixture) was recalcified and placed into the experimental chamber, where the activator for clotting initiation was also placed, and incubated at 37 °С. Fibrin clot formation was detected by imaging light scattering for 30 min (see videos in Supplementary Materials). The following parameters of clot growth were determined based on clotting profiles, calculated by the instrument software: Spontaneous clot formation time, the time required to fill 5% of the analyzed cuvette area with spontaneous clots; and total clot formation time, the time required to fill 100% of the analyzed cuvette area with clots.

### 2.6. Anti-Xa Assay

The assay for quantitative determination of heparin activity (anti-Xa assay) was performed with a Sysmex CA-1500 (Sysmex Corporation, Chuo-Ku, Kobe, Japan) automatic coagulometer and Berichrom^®^ heparin reagent (Siemens Healthcare Diagnostics, Newark, DE, USA). The detection was performed using a kinetic test and the increase in absorbance at 405 nm was recorded. The quantity of unbound Xa was calculated using a calibration curve.

### 2.7. Transmission Electron Microscopy

A 10 μL drop of EVs in PBS was applied to nitrocellulose carbon-coated PELCO^®^ Cu grids (Ted Pella Inc., Redding, CA, USA) and incubated for 1 min. Liquid was removed by touching the paper edge. A 10 μL drop of 2% uranyl acetate was immediately applied to the grid, followed by 15 s incubation and drop removal by touching the paper edge. Samples were examined at 80 kV with a JEM-1011 transmission electron microscope (JEOL, Akishima, Japan) equipped with an Orius™ SC1000 W camera (Gatan Inc., Pleasanton, CA, USA).

### 2.8. Nanoparticle Tracking Analysis

Particle size distributions and number concentrations of isolated EVs were measured with nanoparticle tracking analysis (NTA) using a Nanosight LM10 HS unit (NanoSight Ltd., Amesbury, UK). The instrument contains a 405 nm 65 mW laser unit with no temperature control and high-sensitivity electron multiplying charge-coupled device (EMCCD) Andor Luca camera. All measurements were performed according to the recommendations of the ASTM E2834-12 standard [28]. Briefly, samples were diluted by particle-free PBS up to a final concentration of around 1.5 × 10^8^ particles/mL. Videos of particle Brownian motion were recorded at room temperature with passive temperature readout and the following camera setups optimized for EVs: Camera shutter 850, camera gain 450, lower threshold 715, and higher threshold 10,725. The videos were processed with Nanoparticle Tracking Analysis analytical software version 2.3 build 0033 (NanoSight Ltd., Amesbury, UK) with a detection threshold of 9 multi. At least 12 individual videos, each 60 s, with a total of at least 5000 tracks were recorded and processed. Data from multiple videos were joined together to obtain a particle size histogram and the mean total concentration corrected for dilution factor.

### 2.9. Proteomic Analysis of MSCs and EVs

To avoid contamination of MSCs and EVs with proteins derived from FBS, we additionally washed the sample with PBS, followed by centrifugation. Aliquots (100 μL) of EV suspensions (*n* = 3) were mixed with 20 μL 5 × radioimmunoprecipitation assay buffer (RIPA) lysis buffer containing 250 mM 4-(2-hydroxyethyl)-1-piperazineethanesulfonic acid (HEPES) pH 7.4, 750 mM NaCl, 0.25% SDS, 1.25% sodium deoxycholate, and 2.5% Nonidet P-40, and kept for 30 min at +4 °C. MSCs (*n* = 3) were mixed with 1× RIPA in a ratio of 1:10. Samples were sonicated and centrifuged at 15,000× *g* for 10 min. The total protein concentration of each sample was measured using the Pierce BCA Protein Assay Kit (Thermo Fisher Scientific, Waltham, MA, USA). Proteins from supernatant were precipitated with ice-cold acetone, dissolved in UA solution (8 M urea, 50 mM Tris-HCl, pH 8.5) reduced with DTT 100 mM, and processed according to the filter aided sample preparation (FASP) protocol as described in [29]. The mixture was transferred to a Microcon-10 kDa centrifugal filter unit (MRCF0R010, Millipore, Billerica, MA, USA) and centrifuged at 14,000× *g* for 15 min. Cysteine residues were alkylated by adding 100 μL of UA solution containing 50 mM iodoacetamide to the filter unit, followed by incubation in darkness for 30 min at room temperature. After centrifugation at 14,000× *g* for 10 min, 100 μL of UA solution was added to the filter unit and centrifuged again. This UA washing step was repeated twice, and the filter unit was then washed with 100 μL of 50 mM NH_4_HCO_3_ twice. Next, protein digestion was carried out by adding 40 μL of 50 mM NH_4_HCO_3_ solution containing sequencing-grade trypsin (enzyme-to-protein ratio 1:50) in the filter unit and incubating at 37 °C for 16 h. Digested peptides were eluted by adding 50 μL of ddH_2_O and collected by centrifugation at 14,000× *g* for 15 min as a filtrate, and this step was repeated twice.

Tryptic peptide mixtures were separated on a nano-ESI-HPLC Agilent 1100 system (Agilent Technologies, Santa Clara, CA, USA) using a self-packed capillary column (fused-silica PicoTip emitters, id 75 mm, length 12 cm, nominal tip id 15 mm, filled with Reprosil-Pur Basic C18, 3 mm, 100 A; Dr. Maisch HPLC GmbH, Ammerbuch-Entringen, Germany). The separation was carried out by a 120 min gradient (H_2_O/acetonitrile (ACN) containing 0.1% formic acid (FA)) from 3% to 50% of ACN at a flow rate of 300 nL/min. Mass spectrometry (MS) analysis was carried out by 7 T LTQ-FT Ultra (Thermo Electron, Bremen, Germany) using a nanospray ion source (positive high voltage, 2.1 kV) [30].

Raw MS files were processed with MaxQuant software (version 1.1.1.2) against the SwissProt database 22 with the following parameters: Initial mass tolerance for full MS scans, 7 ppm (parts per million) and 0.5 Da for MS/MS; minimum peptide length for identification, 7 amino acids; match between runs option; 2 or more peptides for protein identification with at least 1 peptide unique for the protein group; fixed modification, carbamidomethylation of cysteines; variable modifications, N-terminal acetylation and methionine oxidation; false discovery rate for proteins and peptides less than 0.01. Label-free analysis was performed using the Perseus software package for protein quantitation. After matrix uploading, reverse proteins and proteins identified only by site were excluded.

Gene Ontology (GO) term enrichment was performed using Perseus software with the SwissProt database.

### 2.10. Flow Cytometry Analysis of Phosphatidylserine Exposure on MSC and EV Fractions

MSC and EV fractions were characterized using a FACS Aria SORP cell sorter equipped with 405 and 355, 488, 561, and 640 nm lasers (BD Biosciences, San José, CA, USA). Staining of MSCs was performed with fluorescein isothiocyanate (FITC)-conjugated annexin V (Sigma-Aldrich, St. Louis, MO, USA) as a marker for phosphatidylserine. Staining of MSC-derived EVs was performed with FITC-annexin V in combination with a PKH26 dye (Sigma-Aldrich, St. Louis, MO, USA) as a marker for lipid membranes. Non-stained MSCs/EVs and single-stained EVs with PHK26 were used as controls. Specimens were sampled for 3 min at a flow rate of 30 µL per min and data were analyzed using Flowing Software (Turku Centre for Biotechnology, Turku, Finland). Since phosphatidylserine is believed to be a marker of apoptotic cells, we analyzed MSCs after serum deprivation for 24 h as a positive control for apoptosis.

### 2.11. Western Blotting

Samples of cell lysates or EVs were loaded on 15% Tris-glycine polyacrylamide gels (10 μg protein per lane). After electrophoresis, gels were blotted onto polyvinylidene difluoride (PVDF) membranes (Amersham Pharmacia Biotech, Amersham, UK). Membranes were blocked with 5% nonfat milk in PBS/0.1% Tween-20 and subsequently incubated with primary mouse antibodies to human TF (PAA524Hu01, 1:1000; Cloud-Clone Corp., Houston, TX, USA) and β-actin (A1978, 1:2000; Sigma-Aldrich, St. Louis, MO, USA). β-actin was used as the loading control. Membranes were processed with secondary antibodies conjugated with horseradish peroxidase 1:10,000 (Imtek, Moscow, Russia). Detection was performed by a ChemiDoc™ MP imaging system (Bio-Rad, Hercules, CA, USA) with a WesternBright™ Enhanced Chemiluminescence kit (Advansta, Menlo Park, CA, USA).

### 2.12. Modulation of MSC Procoagulant Activity by Heparin and Annexin V

For modulation of procoagulant activity by heparin, cells were suspended in PBS containing 5% albumin and unfractionated heparin sodium salt at a concentration of 5, 10, or 50 U/mL (Belpharm, Minsk, Belarus) for 10 min. At the end of incubation, cells were centrifuged at 1600× *g* for 3 min, washed in PBS 1 or 3 times to remove residual heparin, and the pellet was resuspended in 10 µL of PBS. The resultant cell suspensions were then added to blood or plasma obtained from 1 mL of blood. The heparin dose was chosen based on the circulating blood volume according to a 70 kg weight.

An attempt was made to reduce procoagulant activity by masking the phosphatidylserine exposed on MSCs and EVs. MSCs (5 × 10^3^ cells) or EVs (4.3 × 10^8^ particles) were suspended in 90 μL of annexin V-binding buffer, supplemented with 10 mL of annexin V-FITC reagent, and incubated for 30 min. After incubation, cells or EV suspensions were added to 1 mL of whole blood.

### 2.13. Statistical Analysis

Statistical analysis was performed using Statistica 7.0 for Windows (StatSoft Inc., Palo Alto, CA, USA). Values are given as mean ± standard error of the mean (SEM). Variance homogeneity was assessed by Levene’s test. Data were compared using Student’s *t* test when 2 groups were compared and parametric analysis of variance (ANOVA) when more than 2 groups were compared. Differences were considered significant at *p* ≤ 0.05. Welch’s *t*-test with Bonferroni correction was applied to identify significantly changed proteins in the groups studied (*p*-value < 0.05). Hierarchical clustering of the resulting proteins was performed on logarithmized intensities. Heat map analysis was performed for hierarchical clustering of rows and columns to produce a visual representation of the clustered matrix. In this study, Euclidean distance was used for the average clustering method.

## 3. Results

### 3.1. Procoagulant Properties of MSCs against Human Blood

**Figure 1:**
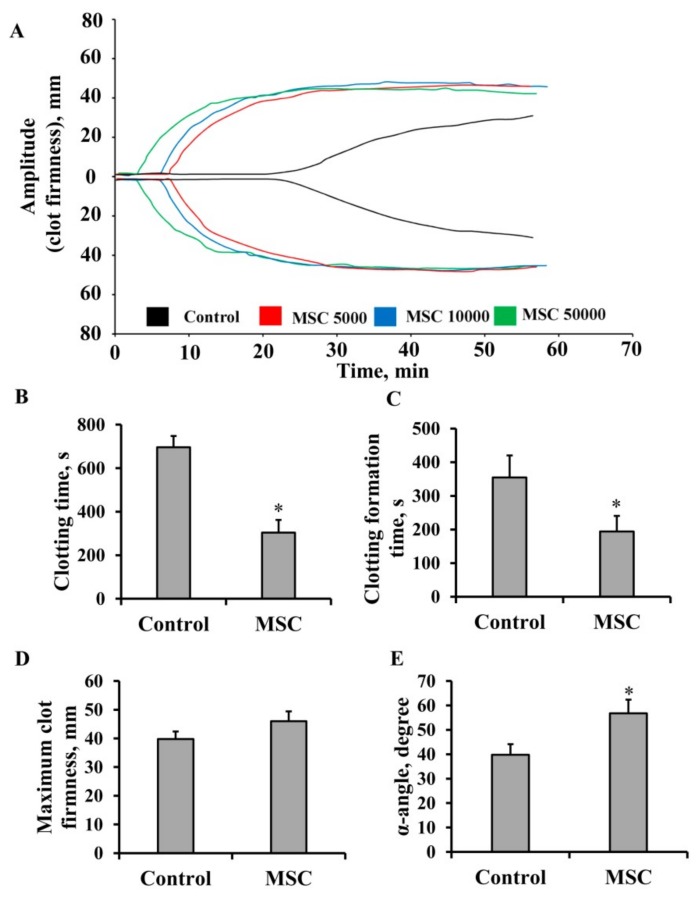
Procoagulant properties of mesenchymal stem cells (MSCs) against human blood. (**A**) Representative thromboelastometry diagram of dose-dependent effects of MSCs on blood coagulation. (**B**) Clotting time, (**C**) clot formation time, (**D**) maximum clot firmness, and (**E**) α-angle assayed by the nonactivated rotational thromboelastometric (NATEM) test. MSCs were resuspended in 1 mL of freshly obtained blood at concentrations of 5 × 10^3^, 1 × 10^4^, and 5 × 10^4^ cells per mL for (A) and 5 × 10^4^ cells per mL for (B–E), then 300 µL of the suspension was sampled for analysis. Data reported as mean ± standard error. Blood from five donors was used, and each blood sample was incubated with MSCs obtained from three different umbilical cord samples. * *p* < 0.05, Student’s *t*-test.

To evaluate the procoagulant properties of human MSCs, we performed NATEM using citrated whole human blood and MSCs. Whole blood samples mixed with MSCs (final concentration 5 × 10^3^, 1 × 10^4^, and 5 × 10^4^ cells/mL) mimicked the in vivo administration procedure. Thromboelastometry diagrams showed dose-dependent hypercoagulation compared to blood without cells (Figure 1A). Thromboelastogram analysis revealed that 5 × 10^4^ cells/mL dramatically promoted blood clotting since CT was decreased 2.3 times, from 696 ± 51 to 304 ± 58 s (Figure 1B, *p* < 0.05), and CFT was decreased 1.8 times, from 350 ± 66 to 194 ± 47 s (Figure 1C, *p* < 0.05). Moreover, the α-angle (which measures the rate of fibrin buildup and cross-linking, hence assesses the rate of clot formation, i.e., thrombin burst) rose from 38 ± 10 to 60 ± 13° compared with control samples (Figure 1E, *p* < 0.05). Another thromboelastogram parameter, MCF (Figure 1D), did not demonstrate significant changes after MSC addition.

**Figure 2:**
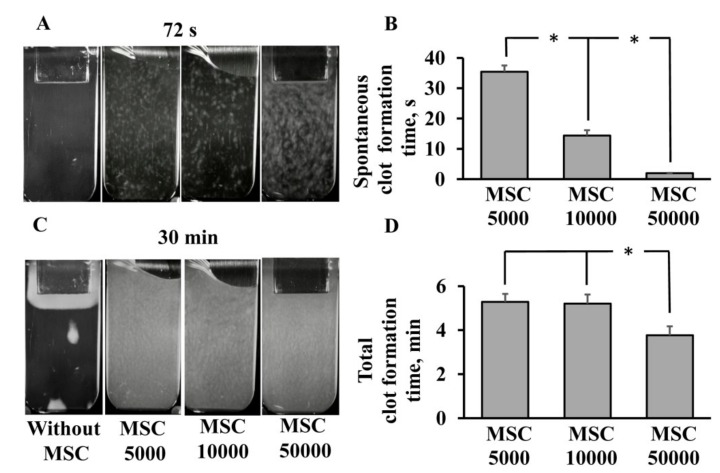
Dose-dependent effect of MSCs on clotting induction in human platelet-free plasma (PFP) using a thrombodynamics test. (**A**) Representative screenshots (at 72 s) of thrombodynamics videos showing spontaneous clot appearance in PFP after the addition of different doses of MSC. (**B**) Spontaneous clot formation time. (**C**) Representative screenshots at the end of assay (30 min) showing the area occupied by a clot in a cuvette. (**D**) Total clot formation time in the full volume of the cuvette. MSC aliquots of 5 × 10^3^, 1 × 10^4^, and 5 × 10^4^ cells and 10 μL of PBS instead of cells (“Without MSC” sample) were resuspended in human PFP obtained from 1 mL of freshly collected blood, then 120 µL of the mixture was sampled for analysis. Note, in the control PFP, spontaneous clot formation did not occur. Data reported as mean ± standard error of the mean (SEM) from at least five experiments. Experiments used the blood from five donors and each blood sample was incubated with MSCs obtained from three different umbilical cord samples. * *p* < 0.05, one-way ANOVA, followed by Tukey’s post hoc analysis.

Next, we studied whether MSCs also possess coagulation properties against platelet-free plasma by using a thrombodynamics assay. This is a method that visually detects fibrin clot growth induced by immobilized TF in a thin layer of unstirred plasma. It represents a more physiologic model of processes occurring in the vessels than conventional clotting assays, as it includes diffusion processes that take place in blood capillaries. In the PFP of healthy donors (control group), the clotting process was initiated on the plate where TF was immobilized and proceeded gradually from this zone to the bottom of the cuvette without any appearance of spontaneous clotting (Figure 2A, Supplementary Video S1). The addition of MSCs to PFP resulted in spontaneous clots appearing across the entire volume of the cuvette at 2 ± 0.1 s of the experiment for an MSC concentration of 5 × 10^4^ cells/mL and at 35 ± 2 s for 5 × 10^3^ MSC/mL (Figure 2A,B). This shows that the rate and quantity of spontaneous clotting were dependent on the cell dose: The higher the MSC density, the faster the clots appeared. Furthermore, we determined the total clot formation time by analyzing 30 min time-lapse videos from the Thrombodynamics Analyzer to evaluate the coagulation process in plasma initiated by MSCs. In the control group, the clot occupied no more than 5% of the cuvette volume and was located near the plate with TF (Figure 2C). The addition of MSCs led to clot formation across the entire volume of the cuvette, and the total clot formation time was 5 min for groups with 5 × 10^3^ and 1 × 10^4^ MSCs per mL, while a higher MSC density (5 × 10^4^ cells/mL) decreased this time to 3 min (Figure 2C,D). This indicates that MSCs have a significant procoagulant property against plasma as well as against whole blood, and these effects are dose-dependent.

### 3.2. Effect of Extracellular Vesicles on Blood Coagulation

MSCs are known to release EVs, such as exosomes and microvesicles, and it is believed that EVs are responsible for at least some of the therapeutic effect of MSCs in a variety of animal models of pathology. Hence, MSC-derived EVs could be used as an alternative to MSC-based therapy in regenerative medicine, so it is important to understand the impact on blood coagulation. To unravel this issue, using NATEM and TD tests, we explored whether EVs could also have procoagulant activity, like MSCs.

**Figure 3:**
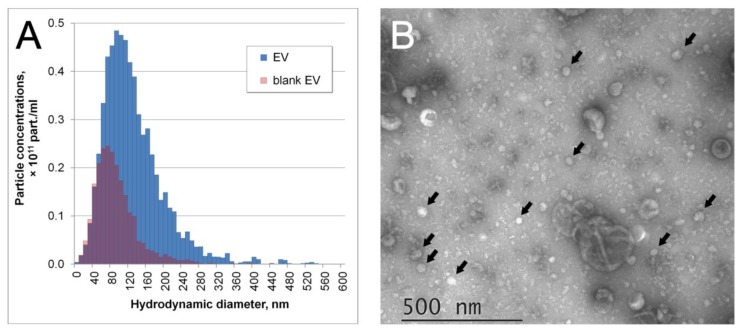
Characteristics of the extracellular vesicle (EV) preparations from conditioned medium after 24 h of MSC cultivation. (**A**) Particle size distributions of EV preparation and resuspended pellet from nonconditioned culture medium passed through all centrifugations (blank EV). (**B**) Transmission electron microscopy image of EV preparation. Arrows mark several smaller particles of nonvesicular morphology.

EVs derived from MSCs were characterized by nanoparticle tracking analysis (NTA), total protein concentration, and transmission electron microscopy (TEM). According to NTA, EVs showed a broad size distribution (Figure 3A), with 90% of the particles between 40 and 250 nm, which indicates the presence of both exosomes and microvesicles. Independently cultured and isolated batches of EVs showed only minor differences in the mean size (132–133 nm) and total particle concentration (6.2–6.8 × 10^11^ particles/mL). Resuspended pellet from nonconditioned culture medium passed through all centrifugations (blank EV) showed the presence of adventitious particles with a mean size of 94 ± 5 nm and total concentration of (2.3 ± 0.2) × 10^11^ particles/mL. Thus, the EV preparations contained around 0.43 × 10^10^ of MSC-derived EVs and 0.23 × 10^10^ of adventitious nanoparticles per μg of total protein. TEM of EV preparations (Figure 3B) confirmed two types of particles. The majority of objects showed a cup-shape morphology characteristic of EVs, with sizes ranging from 40 to 300 nm. A minor fraction of the smaller objects (27–95 nm) had a smooth or angulated shape. 

**Figure 4:**
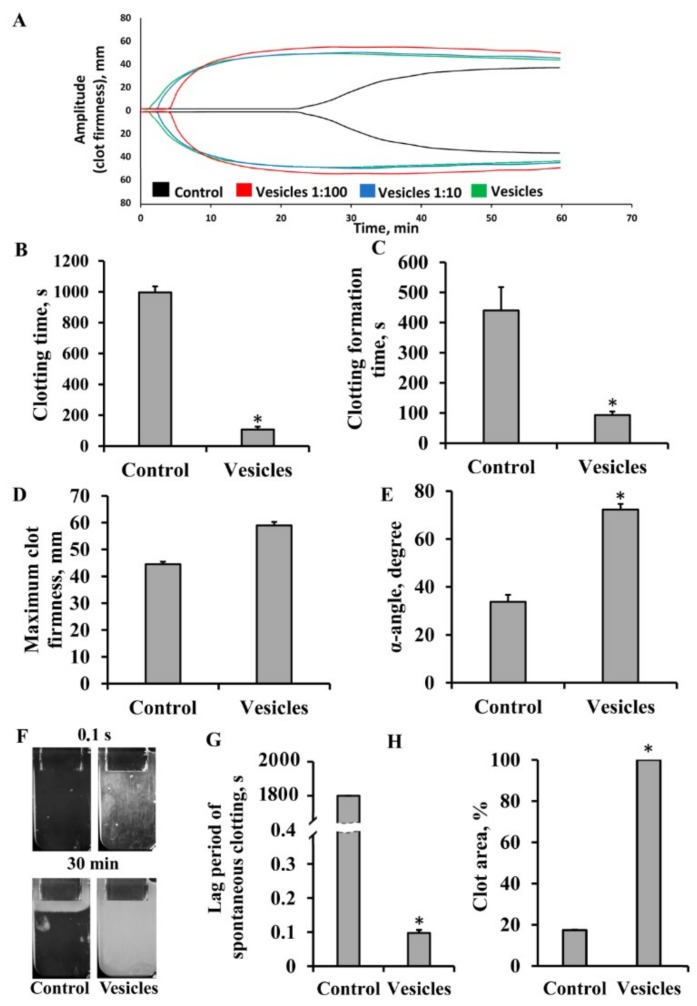
Procoagulant activity of MSC-derived extracellular vesicles in whole blood and platelet-free plasma (PFP). EVs were obtained from conditioned medium of MSCs and added to blood or PFP. Vesicle concentrations were 4.3 × 10^10^ (marked as vesicles), 4.3 × 10^9^ (marked as 1:10), and 4.3 × 10^8^ (marked as 1:100) particles per mL of blood. Data from the NATEM test (A–E) and thrombodynamics (TD) assay (F–H). (**A**) Representative thromboelastometry diagram of dose-dependent effects of EVs on blood coagulation. (**B**) Clotting time, (**C**) clot formation time, (**D**) maximum clot firmness, and (**E**) alpha-angle assayed by the NATEM test. (**F**) Representative screenshots at 0.1 s and 30 min of TD videos showing clotting formation in PFP after addition of EVs. (**G**) Lag period of spontaneous clot formation and (**H**) clot area calculated in % from the full volume of the cuvette at the end of the thrombodynamics test. Blood from five donors was used, and each blood sample was incubated with MSCs obtained from three different umbilical cord samples. Data reported as mean ± standard error of the mean (SEM). * *p* < 0.05, Student’s *t*-test.

The EV preparations at concentrations of 4.3 × 10^10^, 4.3 × 10^9^, and 4.3 × 10^8^ particles/mL caused rapid, dose-dependent activation of blood coagulation when added to citrated whole blood (Figure 4A), while the blank EV sample (ultrafuged pellet from nonconditioned medium) had no effect (Supplementary Figure S2). According to the thromboelastograms, vesicles extremely reduced CT and CFT by nine and four times for blood and plasma, respectively, and increased the α-angle compared to the control samples (Figure 4B,C,E). Similar to MSC administration, we did not find any significant changes of MCFs in samples after EV addition (Figure 4D). Further, we explored the effect of EVs on the coagulation status using the thrombodynamics assay. The lag period of spontaneous clotting decreased dramatically after the addition of EVs, to 0.1 s (Figure 4F,G, Supplementary Video S2). EVs caused a clot to form across the entire volume of the cuvette during the first 15 s, whereas in a similar sample without EVs, the clot occupied only 20 ± 0.3% of the volume at the endpoint of the test (30 min) (Figure 4F,H). These results indicated that MSC-derived EVs, similar to MSCs, had significant procoagulant activity against whole blood and PFP.

### 3.3. Elucidation of Mechanisms Involved in MSC- and EV-Induced Coagulation

**Figure 5:**
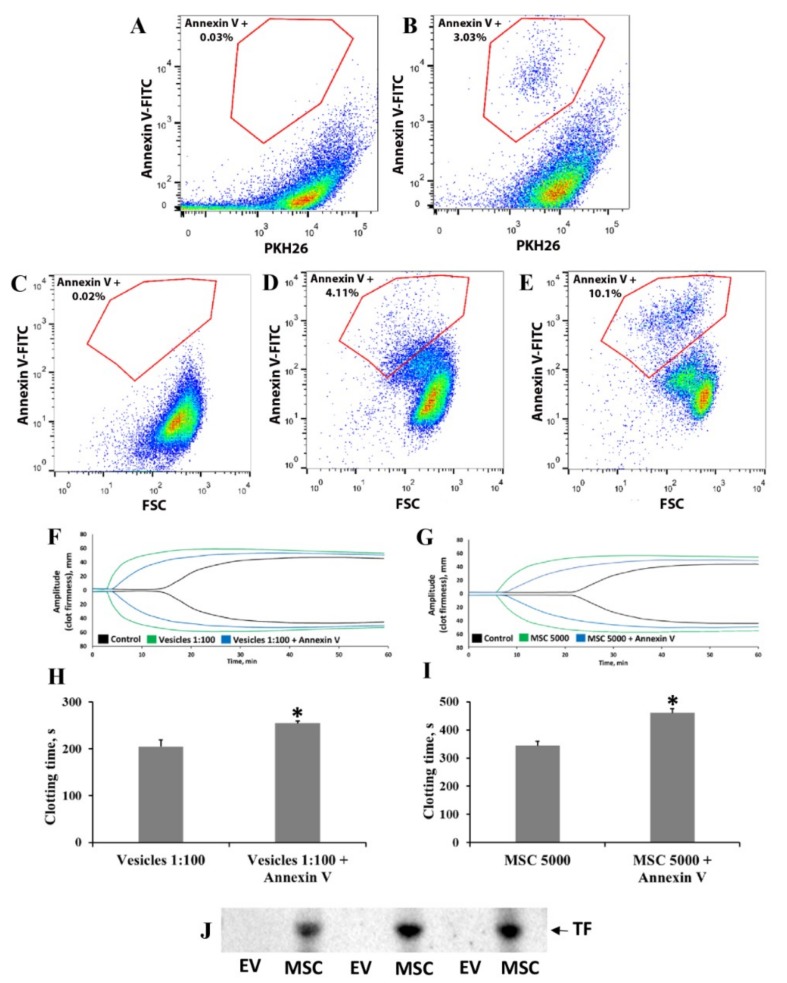
Analysis of phosphatidylserine and tissue factor (TF) expression on MSC and EV surface and the role of phosphatidylserine in procoagulation effects. Elucidation of the mechanisms of MSC- or EV-induced coagulation. MSC-derived EVs were analyzed by flow cytometry with (**A**) PKH26, a lipid-staining dye, or (**B**) a combination of PKH26 and fluorescein isothiocyanate (FITC)-conjugated annexin V. Flow cytometry analysis of phosphatidylserine exposure on the MSC surface using annexin V-FITC: (**C**) unstained MSCs, (**D**) annexin V-FITC stained native MSCs, and (**E**) annexin V-FITC stained MSCs after apoptosis induction. Effect of phosphatidylserine masking on blood coagulation: preincubation of (**F**,**H**) EVs and (**G**,**I**) MSCs with annexin V to shield phosphatidylserine decreased blood coagulation, as shown by thromboelastometry. (**F**,**G**) Representative thromboelastograms and (**H**,**I**) calculated clotting time assayed with the NATEM test. MSCs and EVs were preincubated with annexin V (see Methods), then resuspended in 1 mL of freshly obtained blood and analyzed by the NATEM test. Experiments used blood from three donors and each blood sample was incubated with MSCs from different umbilical cord samples. * *p* < 0.05, Student’s *t*-test. (**J**) Detection of TF on Western blots of MSC and EV samples.

We examined the mechanisms of the procoagulant effects of MSCs and MSC-derived EVs in blood and plasma. A number of studies have demonstrated that the appearance of phosphatidylserine on the outer surface of the cell membrane could be responsible for the induction of a coagulation cascade [18]. Flow cytometry was used to assess the presence of phosphatidylserine-exposing cells and vesicles in MSC culture and EV suspension, respectively. For the detection of EVs, we additionally loaded EVs with PKH26 dye and revealed that among all PKH26-positive particles, about 3% were annexin V-positive (Figure 5A,B). The MSC population showed that about 4% of cells were annexin V-positive (Figure 5C,D). Note, phosphatidylserine exposure in native MSCs seems to not be associated with apoptosis, since apoptotic cells with annexin V-positive staining appeared in areas with more intensive fluorescence (Figure 5E). To assess the contribution of phosphatidylserine to the procoagulant effect of MSCs and EVs, we performed experiments masking phosphatidylserine with annexin V. Preincubation of EVs and MSCs with annexin V increased CT by 25% and 30%, respectively (Figure 5F–I). Moreover, we hypothesized that umbilical MSCs and their EVs could express sufficient amounts of TF on their surface. The presence of TF was analyzed by Western blotting of MSCs and EVs isolated from MSC-conditioned media. We found a significant amount of TF protein in MSC, but not EV samples (Figure 5J).

### 3.4. Analysis of Coagulation-Associated Proteins in MSCs and Extracellular Vesicles

Since blocking of phosphatidylserine only partially diminished coagulation induced by MSCs or EVs, and TF was identified in MSCs only, we proposed additional mechanisms responsible for procoagulant effects. Further, we elucidated the possible mechanisms by which MSCs and MSC-derived EVs could exert procoagulant effects in blood and plasma. The proteomic profiles of MSCs and MSC-derived EVs from six samples revealed 560 proteins (Supplementary Table S2) with a 1% false discovery rate (searched against a decoy database). The mass spectrometry proteomics data have been deposited in the ProteomeXchange Consortium via the PRIDE [31] partner repository with the dataset identifier, PXD012768.

**Figure 6:**
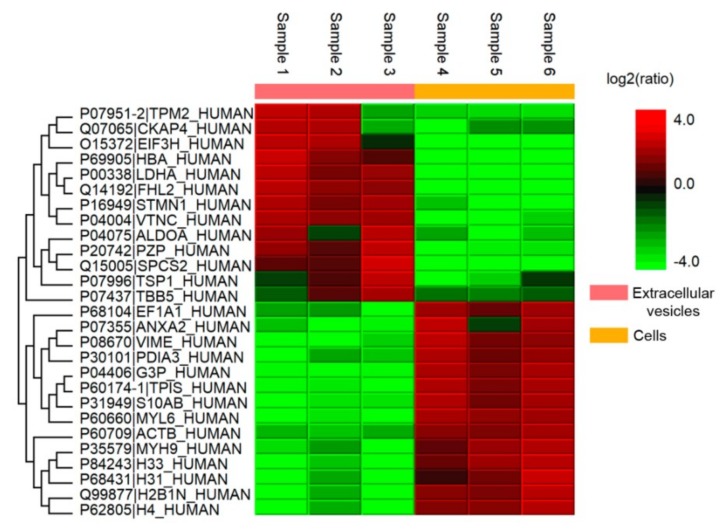
Hierarchical clustering based on label-free comparative analysis of protein expression levels for MSCs and EVs.

Whereas 84 proteins were common for cells and their EVs, 64 proteins were identified only in EVs and 412 only in MSCs. Some EV protein levels substantially differed from those in MSCs: 27 protein levels were significantly changed (*p* < 0.05) according to Welch’s *t*-test with Bonferroni correction for semiquantitative data (Figure 6).

Cells were cultured with 7% FBS, which could influence the proteomic results of MSCs and EVs. To control potential contaminants, an additional search against the Bovine (Bos taurus) and Contaminant databases was performed in MaxQuant software (Supplementary Table S3). The list of coagulation-associated proteins from Table 1 was compared with potential contaminants (Supplementary Table S3) and showed no overlap.

To investigate MSC and EV biological processes, molecular functions, and cellular components, an enrichment analysis was performed using Perseus software. The Gene Ontology biological processes (Supplementary Figure S3) significantly represented (*p* < 0.05) in the EV proteome involved the following: cellular component organization (29%, including protein complex assembly, nucleosome assembly, chromatin assembly or disassembly, protein polymerization): Platelet activation (6%), platelet degranulation (4%), tissue migration (5%), phagocytosis (5%), mesenchyme morphogenesis (4%), and mesenchyme development (5%). From a biological perspective, these proteins are functionally involved in angiogenesis, blood coagulation, extracellular matrix remodeling, the inflammatory response, and apoptosis. A similar pattern was observed in whole cells, although the total number of detected proteins and gene ontology processes was higher (335 proteins with 289 unique ones), and the signal pathways associated with coagulation were also presented: Platelet activation (10%), platelet degranulation (7%), platelet aggregation (3%), blood coagulation (12%). The Gene Ontology molecular functions (Supplementary Figure S4) enriched in the EV proteome were represented almost exclusively by binding processes (52%), as well as in the MSC proteome (87%). Finally, the major Gene Ontology cellular components (Supplementary Figure S5) were divided into two parts: Intracellular origin (49%, membrane-bounded organelle, intracellular organelle, macromolecular complex, nucleus) and extracellular origin (42%, mainly EVs). In total, we found 22 proteins associated with blood clotting (identified as the blood coagulation category in Gene Ontology) and represented in either EVs or MSCs or both proteomes (Table 1).

### 3.5. Procoagulant Activity of MSCs in Patients Undergoing Anticoagulant Therapy with Heparin

**Figure 7:**
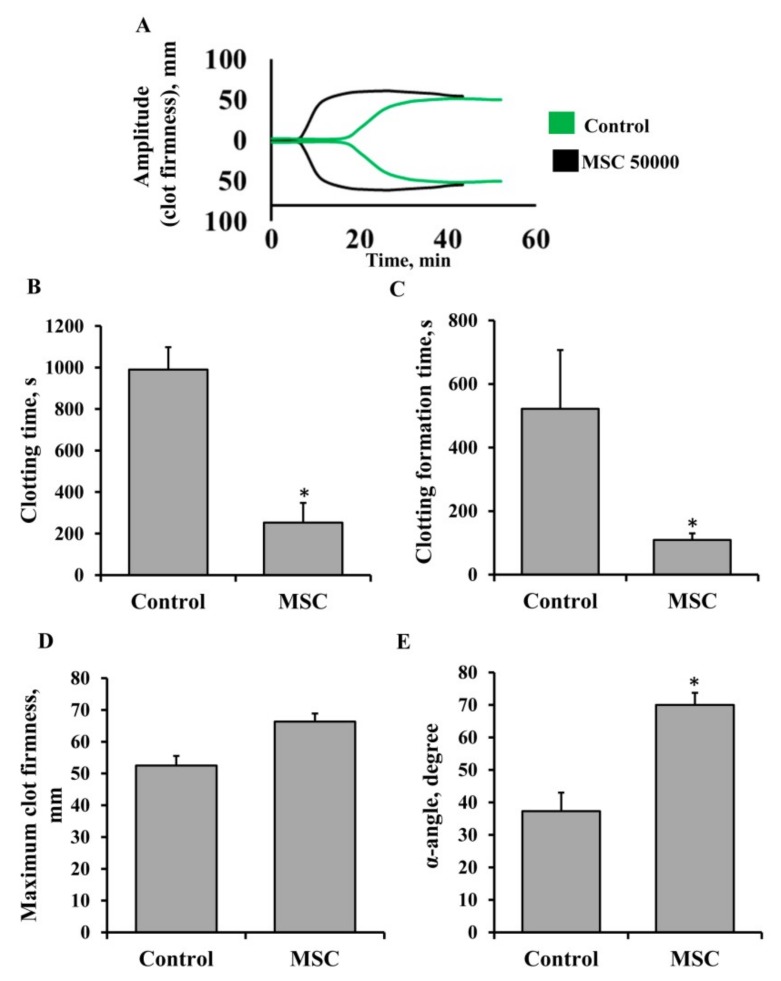
Procoagulant effects of MSCs in the whole blood of patients treated with heparin. (**A**) Representative thromboelastometry diagrams of clotting in whole blood from donors show a change in the clot elasticity over time depending on the addition of MSC to the sample. (**B**) Clotting time, (**C**) clot formation time, (**D**) maximum clot firmness, and (**E**) α-angle assayed by the NATEM test. MSCs were resuspended in 1 mL of freshly obtained blood at a concentration of 5 × 10^4^ cells per mL, then 300 µL of the suspension was sampled for analysis. Blood from seven patients was used, and each blood sample was incubated with MSCs from three different umbilical cord samples. * *p* < 0.05, Student’s *t*-test.

Next, our goal was to assess whether the presence of an anticoagulant, heparin, in the blood of patients would affect the procoagulant effects of MSCs. Blood samples were collected from seven newborn babies receiving heparin treatment due to pathologies of hemostasis. The therapeutic dose of heparin was 5 U/h/kg for all patients. We performed a NATEM test using whole blood samples (Figure 7) to evaluate the effect of MSCs on clotting. We found that MSCs promoted clot formation in the whole blood of these patients in a similar manner to the blood of healthy donors (compare Figure 7A to Figure 1A). The addition of MSCs to whole blood at a dose of 5 × 10^4^ cells/mL decreased CT from 990 ± 108 to 252 ± 95 s and CFT from 522 ± 184 to 109 ± 21 s (Figure 7B,C) and increased the α-angle by 53% (Figure 7E), whereas MCF did not change (Figure 7D). It should be noted that the value of anti-Xa activity in all patients was 0.17 ± 0.02 U, which confirms the presence of heparin in the blood.

### 3.6. Modulation of MSC Procoagulant Activity by Heparin

**Figure 8:**
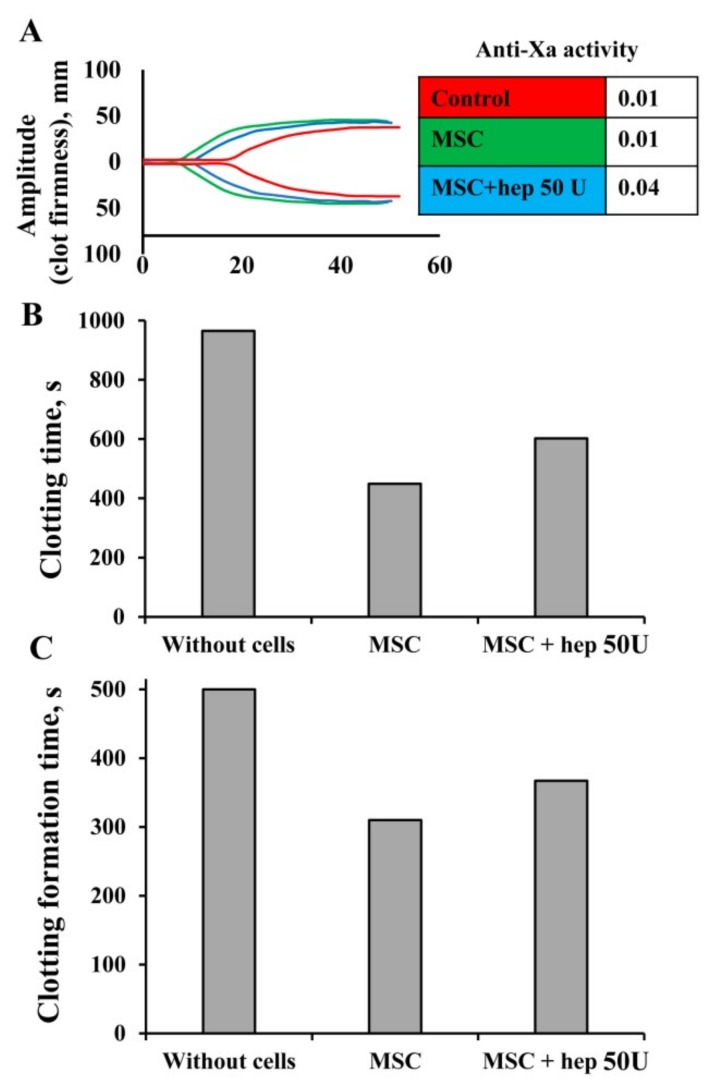
Modulation of MSC procoagulant activity by heparin pretreatment in vitro. (**A**) Representative diagram of thromboelastometry. (**B**) Clotting time and (**C**) clot formation time assayed by the NATEM test. MSCs were pretreated with 50 U heparin for 10 min, followed by washing three times in PBS. Further, MSCs were added to 1 mL of freshly obtained blood, reaching a final concentration of 5 × 10^4^ cells per mL, then 300 µL of the suspension was sampled for analysis.

Since experiments with the blood from patients undergoing heparin therapy showed that heparin in the blood in vivo did not prevent the procoagulant effect of MSCs, we tested the possibility of blocking the procoagulant action of MSCs by treatment with heparin in vitro. We hypothesized that pretreatment of MSCs with heparin could alleviate their procoagulant effect by blocking TF on the cell surface. We pretreated MSCs with doses of unfractionated heparin from 1 to 50 U for 10 min, followed by washing one or three times with PBS. MSCs were added to 1 mL of whole blood with a subsequent NATEM test. Simultaneously, we evaluated the levels of free heparin in samples using an anti-Xa activity test. We found that in MSC samples exposed to a single wash, clotting was completely abrogated and anti-Xa activity for 50 U was 0.96 U, pointing to a high residual concentration of heparin (data not shown). In contrast, if MSCs were washed three times, their procoagulant property was slightly attenuated (Figure 8A). After such MSC treatment with heparin, the CT increased by 16% and the CFT by 11% according to the thromboelastogram (Figure 8B,C). In these samples, anti-Xa activity was 0.04 U, indicating the absence of free heparin (Figure 8A).

## 4. Discussion

In this study, we showed that MSCs isolated from the Wharton jelly of postpartum umbilical cords have innate procoagulant activity. In the last decade, the umbilical cord has become an increasingly used source of MSCs for preclinical and clinical studies [32,33]; thus, the full spectrum of biological properties of these cells should be explored, specifically the recently described procoagulatory effects. Furthermore, MSC-derived EVs, which include exosomes and microvesicles, are being examined for their application in MSC-based cell therapy [34,35]. However, there is not enough evidence for the safety or evaluation of the potential risks of using EVs in clinical practice [36,37]. It is known that EVs could carry on their surface the same receptors and proteins as parental cells, which has been described for EVs from MSCs [38] or cancer cells [39,40]. Considering the detected effect of MSCs on blood clotting, EV therapy could also have the same risk of thromboembolism as is known for MSC therapy. Indeed, in our work, we demonstrate that EVs as well as parental MSCs possess procoagulant properties in vitro, accelerating the clotting time and clot formation time and increasing the α-angle. It is clearly seen by thrombodynamics assay that the addition of EVs and MSCs to platelet-free plasma leads to the spontaneous formation of fibrin clots in a few seconds, followed by complete clot formation within a few minutes, whereas such spontaneous clotting was absent without added cells. While procoagulant activity has not been previously reported for MSCs isolated from the umbilical cord, our data are similar to previously obtained results on the procoagulant effects of MSCs from bone marrow [17], adipose tissue [8], and placental decidua [41]. Moreover, we revealed that EVs isolated from the MSC-conditioned medium also have procoagulant properties.

Note that in the field of cell technologies, the phenomenon of MSC procoagulant activity has been mentioned only recently, whereas in oncology, the problem of procoagulant activity of tumor cells and their EVs was known much earlier and a number of mechanisms have been proposed to explain such observations (see review [42]). We believe that a number of these mechanisms may have a similar nature to the procoagulant activity of MSCs. Tumor cells and tumor-derived vesicles were shown to express TF which interacts with blood/plasma thus recruiting FVIIa, and leading to the formation of the extrinsic tenase complex [43,44,45,46]. Another mechanism involves an exhibition of procoagulant lipids, particularly phosphatidylserine, on the cell surface [47,48]. It has been described that MSC-derived EVs can expose phosphatidylserine on their surface [49], which provides a catalytic surface for the formation of the tenase (factors VIIIa, IXa, and X) and prothrombinase (factors Va, Xa, and II) complexes of the coagulation cascade. Phosphatidylserine may additionally contribute to the transformation of TF from its inactive, encrypted form into a biologically active state [18,50]. In this study, we showed that only a part of MSC and EV populations (3–4%) were annexin V-positive, meaning that these objects had phosphatidylserine on their surface. Preincubation of MSCs or EVs with annexin V before running the NATEM test showed a decreased clotting time by 25–30% vs. that in untreated MSCs/EVs. Thus, our experiments confirmed phosphatidylserine involvement in the procoagulant effects caused by MSCs and their EVs. However, since shielding phosphatidylserine with annexin V resulted in abrogation of only part of the procoagulant effect, this provides evidence for some alternative mechanisms activated by MSCs. In addition, using Western blot technique, we analyzed MSCs and EVs for the presence of a tissue factor, which is another important coagulation element. Tissue factor was revealed in MSCs but not in derived vesicles, which supports that MSC-induced coagulation may be TF-mediated, but TF is not relevant to the procoagulant effect of EVs. Previous investigations have documented the procoagulant activity of MSCs from different sources, associated with the expression of TF [8,15,16,17,41,51]. Recently, Tripisciano and colleagues investigated the potential of platelet- or monocyte-derived EVs to support thrombin generation and concluded that EVs support the propagation of coagulation irrespective of the cell type via the exposure of phosphatidylserine, while the expression of TF on EVs appears to be limited to pathological conditions [52]. Thus, the authors suggest that in this case, the primary effector of the coagulatory cascade is phosphatidylserine rather than TF.

During a search for potential procoagulant proteins, we performed a proteome analysis of MSCs and EVs, which revealed a number of proteins associated with procoagulant activity, such as coagulation factor V, prothrombin, myosin-9, histones, and CD9. It is well known that these proteins could regulate coagulation through platelet-dependent mechanisms [53,54,55,56]. СD9 is one of the most abundant membrane proteins of EV: It induces platelet activation [57], promotes platelet aggregate stability, and enhances fibrinogen binding [58]. CD9-deficient mice show impairment in blood coagulation, while CD9 knockout appears to prevent excessive thrombus growth, but does not appear to play a critical role in primary hemostasis [59]. Notably, in our study, EVs possess strong procoagulation activity in platelet-free plasma. It is remarkable that according to our data, EVs contain annexin V, which is known for its anticoagulant properties [60] by binding with phosphatidylserine [61,62], and thus we can speculate that the procoagulant properties of EVs depend on a balance between the activities of pro- and anticoagulant proteins. However, the procoagulant activity of EVs through platelet-dependent mechanisms should be taken into account in clinical practice, particularly in patients with hemostasis abnormalities. Eirin et al. also found in MSC-derived EVs a robust expression of proteins involved in blood coagulation, such as von Willebrand factor, coagulation factor X, and plasma kallikrein, which may mediate the innate procoagulant activity of MSCs [63]. Recently, it was reported that thrombin-dependent activation of platelet protease-activated receptor (PAR) expressed on the MSC surface can also contribute to blood clotting [64] through fibronectin production by MSCs. Thus, MSCs and EVs could have different patterns of procoagulant activity, and the exact pathways of these effects remain to be explored.

When describing the mechanisms of the procoagulant effects of MSCs, it should be taken into account that a number of confounding factors, such as the cell dose, passage number, and viability, affect the triggering of the coagulation cascade by MSCs [8,15,65]. Our study demonstrates dose-dependent effects of MSCs on blood clotting in vitro. We used cell concentrations from 5 × 10^3^ to 5 × 10^4^ cells/mL, which correspond to the most commonly used therapeutic doses in preclinical and clinical practice [60]. The greatest procoagulant effect was observed at a dose of 5 × 10^4^ cells/mL. This is important for clinical use, as the therapeutic effects could be enhanced by increasing the number of injected cells (up to 12 × 10^6^ cells/kg) [66]. It means that with an increased therapeutic dose, the risk of thrombosis potentially increases as well. Our choice of EV concentration was also based on experimental animal studies that used 30–50 µg of total EV protein for the rat/mouse, which was sufficient for the manifestation of therapeutic effects [67,68]. We added EVs containing 10 µg of total protein (corresponding to ~1 × 10^7^ cells) per 1 mL of whole blood and detected a significantly reduced CT and CFT. Using the thrombodynamics assay, we found that the addition of vesicles caused a decreased lag period of spontaneous clotting to about 0.1 s, which was 20 times shorter than that of MSCs. This result shows that vesicles possess stronger procoagulant properties than MSCs themselves.

Another important factor determining the procoagulant activity of MSCs is the passage number. In this study, we used MSCs and MSC-derived EVs only at the third passage as the most preferred for the purpose of clinical use [69]. Several studies have shown that increasing the passages of MSC cultivation from 3 to 12 correlates with an increased expression of TF [17,19].

It should be noted that in our experiments, intravenous transplantation of MSCs at a dose of 12 × 10^6^ cells per neonatal rat did not cause any animal deaths or signs of thromboembolism (Supplementary Table S1). In contrast, the study by Tatsumi et al. described that within 24 h after the injection of adipose-derived MSCs at a dose 5 × 10^6^ cells/kg, the mortality rate of the mice was nearly 85%. Histological assessment of these mice revealed multiple fibrin clots formed in the right ventricle and pulmonary arteries [8]. In the work of Liao et al., death of the animals during the first hour after transplantation was only observed with very high doses, 8–12 × 10^7^ cells/kg, in systemic infusion of bone marrow-derived MSCs. Histological analysis showed marked microthrombus formation in microvessels and arterioles of the lung, heart, liver, kidney, and spleen [17]. Thus, studies of the acute adverse effects of MSCs used quite different cell dosages, which may be due to both the differences in cell features and the animals used. It is also important to consider that the concentration of cells in the body depends on the organ. It is well known that after systemic administration, most cells in the first minutes are redistributed into the lungs due to the effect of the “vascular trap,” when MSCs get stuck in the lung capillaries [70,71] and could cause pulmonary thrombosis [52]. Thus, the number of MSCs in the lungs may exceed the concentration in other organs by several times, increasing the risk of thromboembolism. Indeed, all clinical reports of the adverse effects of MSCs on hemostasis were associated with the lungs [8,10,11].

It should be noted that we cannot exclude the possibility that the observed effects of the procoagulant activity of MSCs represent an artifact of the in vitro system, which does not include, for example, hemodynamics. A number of studies revealed no side effects or animal deaths from thromboembolism during MSC-based therapy. On the other hand, the described rare clinical cases of thromboembolism [8,9,10,11] associated with MSC transplantation require more detailed analysis. It can be assumed that patients with hemostatic disorders represent a risk group who may develop side effects associated with procoagulant MSC activity. In a recent clinical study, it was shown that transplantation of MSCs at a dose of 1 × 10^6^ or 4 × 10^6^ cells/kg with simultaneous systemic administration of lipopolysaccharides (2 ng/kg) resulted in a dose-dependent increase in the D-dimer level in the blood [12].

One approach to minimize the potential procoagulant side effect of MSCs is treatment with heparin. We are the first to use the blood samples of patients receiving heparin to study the procoagulant properties of MSCs. However, regardless of the presence of heparin (confirmed by anti-Xa assay), procoagulant activity of MSCs was still observed in the blood. Thromboelastometry parameters of heparin-treated patients were similar to those of healthy donors without heparin. Thus, we can conclude that therapeutic doses of unfractionated heparin do not abolish the procoagulant effects observed in vitro, and the use of heparin to abolish excessive procoagulant activity of MSCs in clinical practice may not be efficient. However, a number of animal studies have shown the high efficacy of heparin in preventing procoagulant activity of MSCs [8,17,51]. We assume that these studies used very high doses of heparin unrelated to clinical practice. Indeed, in our work, after the heparin treatment of MSCs, the presence of high anti-Xa activity and clotting was completely abrogated, pointing to excessive amounts of free heparin. It is known that heparin can be absorbed by the cell surface [72,73]; however, we were unable to confirm the efficiency of absorbed heparin in preventing the procoagulant activity of MSCs. In our experiments, triple washing of cells from heparin resulted in only slight increases in the CT and CFT. The fact that different proteins are involved in the procoagulant effects of MSCs and EVs may explain the lack of effectiveness of heparin monotherapy. Stephenne and colleagues demonstrated that only a combination of heparin and bivalirudin (direct thrombin inhibitor) caused a decrease in MSC-induced clotting. The authors supposed that to control the procoagulant activity of MSCs, a combination of anticoagulants would be needed [19].

## 5. Conclusions

We demonstrated that umbilical MSCs and extracellular vesicles derived from them have a reasonably high procoagulant potential. Therapeutic doses of unfractionated heparin in patients’ blood (administered in vivo) do not abrogate the procoagulant properties of MSCs. In this regard, it is necessary to unravel the molecular mechanisms of MSC-mediated coagulation in detail as well as the ways to prevent these processes. Thus, it is necessary to establish exclusion criteria for patients with a high risk of adverse effects of MSC therapy, which may be based on an analysis of hemostasis parameters and some personalized tests on the compatibility of blood with transplanted MSCs.

## Figures and Tables

**Table 1:** MSC and EV proteins associated with blood coagulation.

Gene Name	UniProt ID	Protein Name	Normalized Intensity	
			MSC	EV
YWHAZ	P63104	14-3-3 protein zeta/delta	1.3 × 10^4^	2.2 × 10^5^
ACTB	P60709	Actin, cytoplasmic 1	9.9 × 10^6^	8.4 × 10^7^
ACTG1	P63261	Actin, cytoplasmic 2	9.9 × 10^6^	5.1 × 10^7^
A2MG	P01023	Alpha-2-macroglobulin	6.9 × 10^6^	0
ACTN1	P12814	Alpha-actinin-1	0	4.6 × 10^5^
ANXA5	P08758	Annexin A5	0	4.9 × 10^5^
CD59	P13987	CD59 glycoprotein	4.1 × 10^5^	1.8 × 10^4^
CD9	P21926	CD9 antigen	2.1 × 10^5^	4.7 × 10^4^
FA5	P12259	Coagulation factor V	7.2 × 10^4^	0
CSRP1	P21291	Cysteine and glycine-rich protein 1	0	1.5 × 10^4^
FLNA	P21333	Filamin-A	0	3.7 × 10^6^
GNB1	P62873	Guanine nucleotide-binding protein G(I)/G(S)/G(T) subunit beta-1	1.4 × 10^4^	2.9 × 10^3^
HSPB1	P04792	Heat shock protein beta-1	0	2.1 × 10^5^
HBB	P68871	Hemoglobin subunit beta	3.0 × 10^5^	0
HIST1H3A	P68431	Histone H3.1	1.0 × 10^5^	1.8 × 10^6^
HIST2H3A	Q71DI3	Histone H3.2	1.1 × 10^4^	8.8 × 10^5^
H3F3A	P84243	Histone H3.3	1.6 × 10^4^	4.4 × 10^5^
MYL12A	P19105	Myosin regulatory light chain 12A	0	3.2 × 10^5^
MYH9	P35579	Myosin-9	2.0 × 10^5^	4.9 × 10^6^
THRB	P00734	Prothrombin	7.8 × 10^5^	0
TLN1	Q9Y490	Talin-1	1.5 × 10^4^	1.3 × 10^5^
VCL	P18206	Vinculin	0	3.5 × 10^5^

## Supplementary Materials

The following are available online: Figure S1. Phenotyping of MSC. Figure S2. Influence on blood coagulation of a pellet from nonconditioned culture medium passed through all centrifugation procedures (blank EV). Figure S3. Gene Ontology (GO) enrichment diagrams of MSC-derived extracellular vesicle specific proteome: biological processes. Figure S4. Gene Ontology (GO) enrichment diagrams of MSC-derived extracellular vesicle specific proteome: molecular functions. Figure S5. Gene Ontology (GO) enrichment diagrams of MSC-derived extracellular vesicle specific proteome: cellular components. Table S1. Survival of pups after intravenous administration of MSC. Table S2. Mass spectrometry proteomics data. Table S3. List of potential contaminants. Video S1. Dose-dependent effect of MSC on clotting induction in human platelet-free plasma using thrombodynamics test. Video S2. Effect of EVs on clotting induction in human platelet-free plasma using thrombodynamics test.
