# Supplementary material for: Effect of MSCs and MSC-Derived Extracellular Vesicles on Human Blood Coagulation
Source: Cells. 2019 Mar 19;8(3):258. doi: 10.3390/cells8030258 (PMC6468445; doi:10.3390/cells8030258)
Supplement: Supplementary file 1 [file cells-08-00258-s001.zip › Supplemental files/SUPPLEMENTAL FIGURES.pdf]

SUPPLEMENTAL MATERIALS and METHODS

MSC phenotyping

To analyze the cell-surface expression of typical protein markers, adherent cells were incubated with trypsin-EDTA solution 0.25% (Paneco, Moscow, Russia). The cells were washed in PBS and incubated for 10 minutes in commercial MSC phenotyping kit (Miltenyi biotec, Germany) with the following monoclonal anti-human antibodies: CD90-FITC (clone: DG3, isotype: mouse IgG1), CD105-PE (clone: 43A4E1, isotype: mouse IgG1), CD73-APC (clone: AD2, isotype: mouse IgG1), CD45-PerCP (clone: 5B1, isotype: mouse IgG2a), CD14-PerCP (clone: TÜK4, isotype: mouse IgG2a), CD20-PerCP (clone: LT20.B4, isotype: mouse IgG1), CD34-PerCP (clone: AC136, isotype: mouse IgG2a); isotype controls CD73-APC (clone: AD2, isotype: mouse IgG1), CD90-FITC (clone: DG3, isotype: mouse IgG1), CD105-PE (clone: 43A4E1, isotype: mouse IgG1). After the incubation $1*10^5$ labeled cells were analyzed using a FACS CANTO II flow cytometer (BD Biosciences, USA).

MSC infusion into rat's pups

The animal protocols used in this work were evaluated and approved by the institutional animal ethics committee at A.N. Belozersky Institute of Physico-Chemical Biology in accordance with FELASA guidelines. The experiments were performed on outbred white rats. The animals were obtained from the animal facility of the A.N. Belozersky Institute of Physico-Chemical Biology. To determine the possible acute toxicity of MSC, seven-day-old pups (15.6 ± 1.8 g) from four litters were equally and randomly divided into the following experimental groups : PBS treated group and groups of pups that received 1, 6 or $12 \cdot 10^6$ MSC per kilogram (n=6 for each group). The cultured MSC were harvested as a single cell suspension by trypsinization, re-suspended and infused into pups at a volume of 100 µl via jugular vein under isoflurane anesthesia. The pups were observed every hour for 6 hours to evaluate the acute toxicity (dyspnea and cyanosis). Mortality was assessed 24 h after MSC transplantation.

SUPPLEMENTAL FIGURES

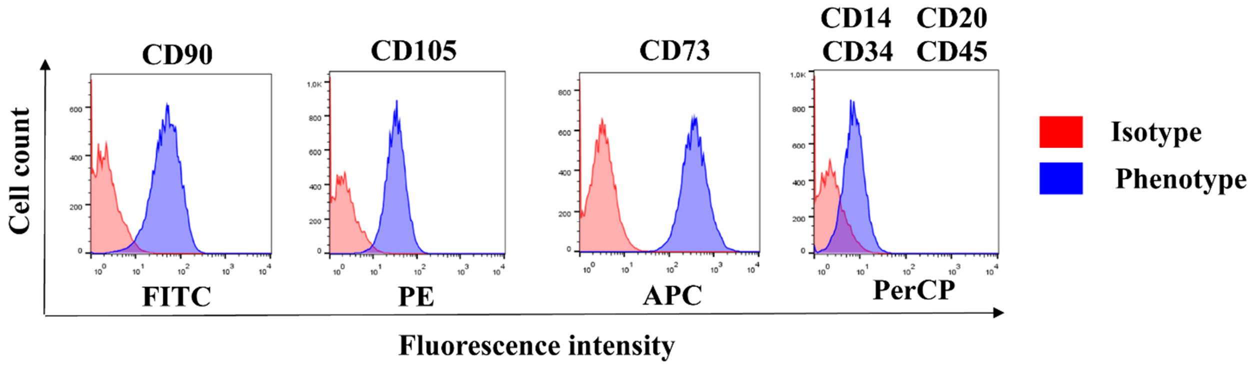

Figure S1. Phenotyping of MSC. Cells were cultured to the third passage, trypsinized, incubated with specific fluorescent antibodies against cell surface markers and analyzed using flow cytometry method. The representative histograms of cell surface markers expression are depicted.

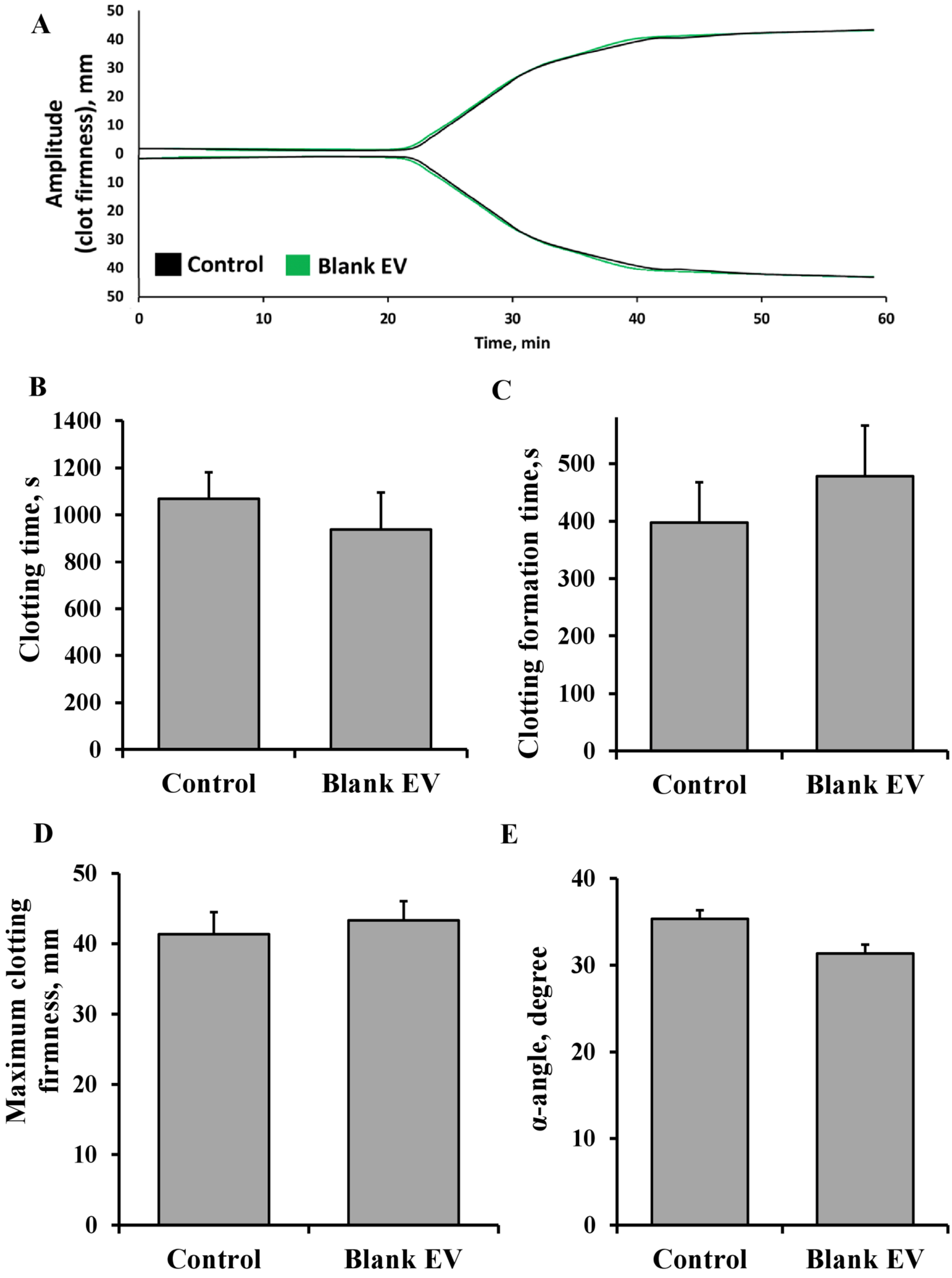

Figure S2. Influence on blood coagulation of a pellet from nonconditioned culture medium passed through all centrifugation procedures (blank EV). (A) A representative thromboelastometry diagram , (B) Clotting time, (C) clot formation time, (D) maximum clot firmness and (E) α-angle assayed by NATEM test. Blood from five donors was used.

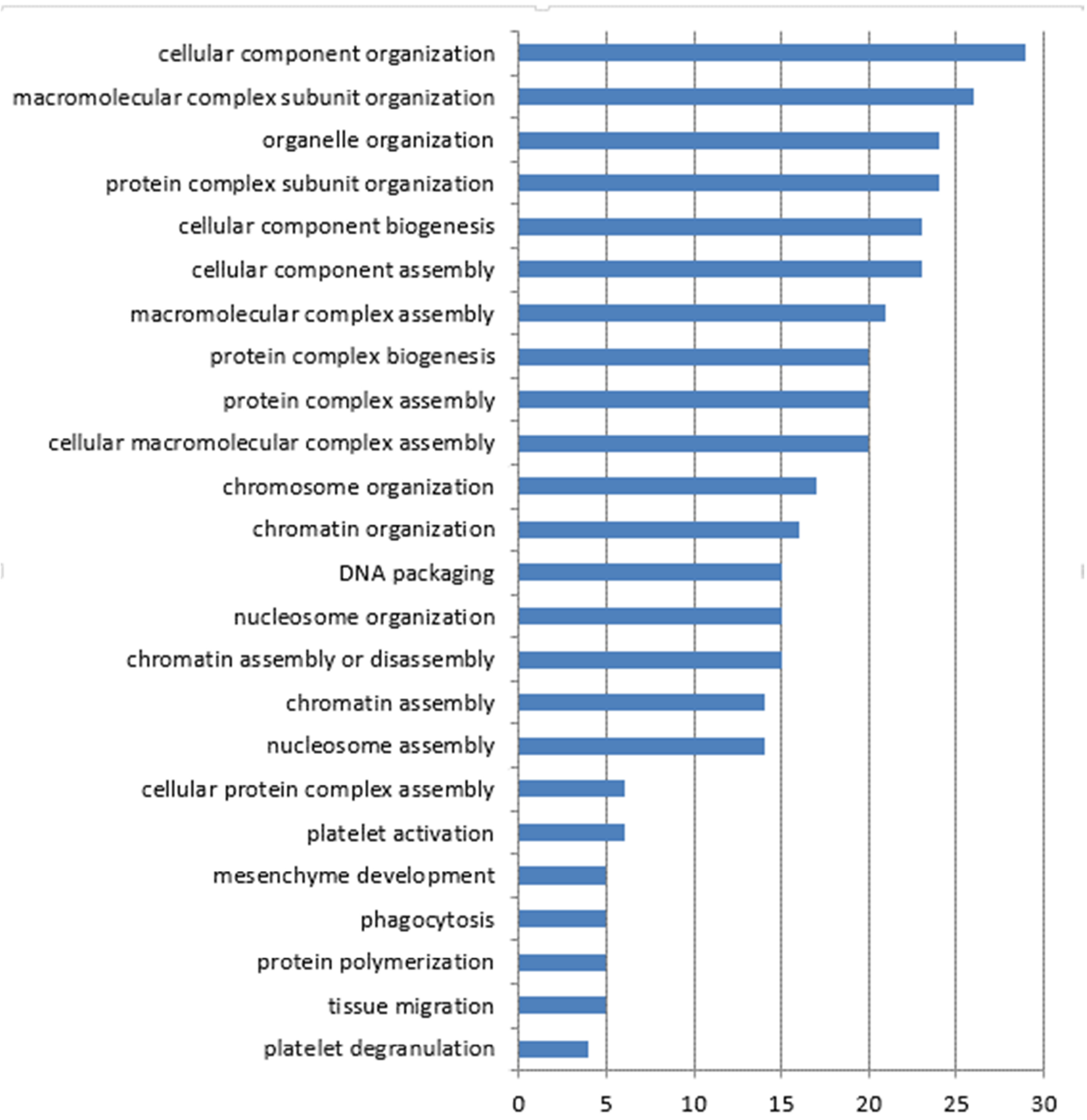

Figure S3. Gene Ontology (GO) enrichment diagrams of MSC-derived extracellular vesicle specific proteome: biological processes.

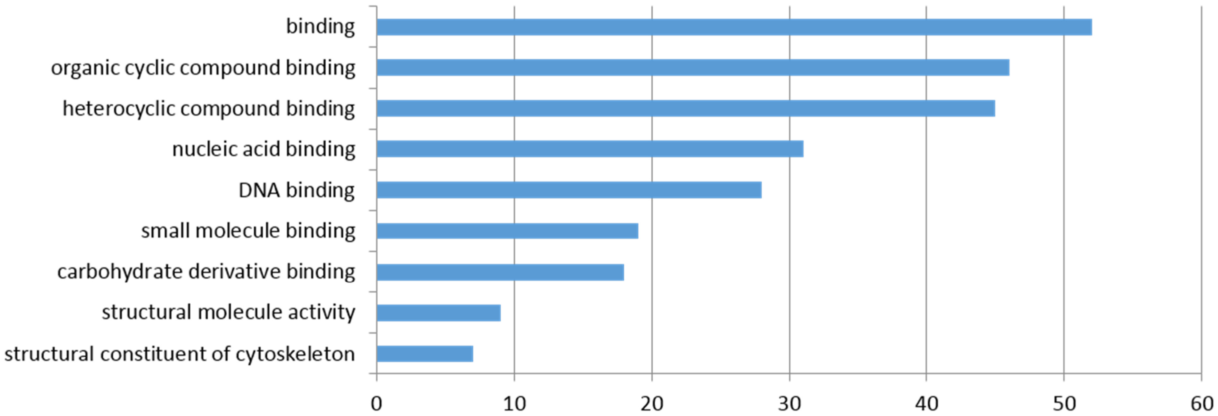

Figure S4. Gene Ontology (GO) enrichment diagrams of MSC-derived extracellular vesicle specific proteome: molecular functions.

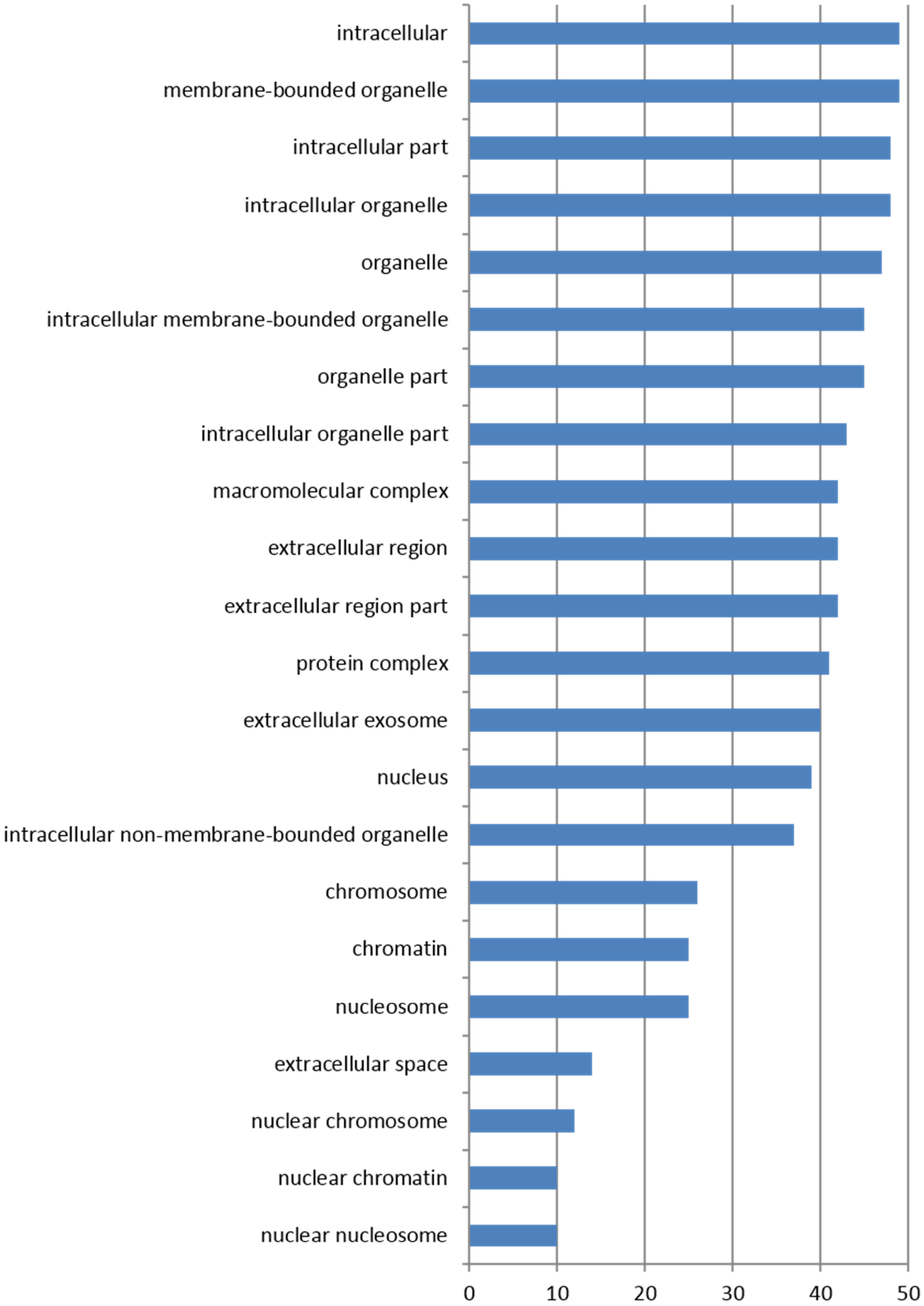

Figure S5. Gene Ontology (GO) enrichment diagrams of MSC-derived extracellular vesicle specific proteome: cellular components.

SUPPLEMENTAL TABLE

Table S1. Survival of pups after intravenous administration of MSC.

	PBS	$1*10^6$ MSC per kg	$6*10^6$ MSC per kg	$12*10^6$ MSC per kg
	n=6	n=6	n=6	n=6
Mortality	0%	0%	0%	0%
Dyspnea	0%	0%	0%	0%
Cyanosis	0%	0%	0%	0%
